# Validation of a Newly Developed Assessment Tool for Point-of-Care Ultrasound of the Thorax in Healthy Volunteers (VALPOCUS)

**DOI:** 10.3390/tomography11090097

**Published:** 2025-08-26

**Authors:** Patrick Hoffmann, Tobias Hüppe, Nicolas Poncelet, Julius J. Weise, Ulrich Berwanger, David Conrad

**Affiliations:** 1 Department of Anaesthesiology, Intensive Care and Pain Therapy, Saarland University Medical Center and Saarland University Faculty of Medicine, 66424 Homburg, Saarland, Germanytobias.hueppe@uks.eu (T.H.);; 2 Department of Anesthesiology, Waterford University Hospital, X91 ER8E Waterford, Ireland; 3 Institute for Medical Biometry, Epidemiology and Medical Informatics, Saarland University Medical Center, 66424 Homburg, Saarland, Germany; juw@med-imbei.uni-saarland.de; 4 Marienhaus Clinic St. Elisabeth Saarlouis, Department of Anaesthesiology, Intensive Care and Pain Therapy, 66740 Saarlouis, Saarland, Germany; ulrich.berwanger@marienhaus.de

**Keywords:** ultrasound, point of care ultrasound, POCUS, assessment tool, validation, lung ultrasound

## Abstract

Objectives: Point-of-care ultrasound (POCUS) has become an integral part of emergency, intensive care, and perioperative medicine. However, the training and subsequent evaluation of POCUS users are still not standardized. The aim of the study was to develop and validate an assessment tool for POCUS users. Methods: After reviewing the existing literature and a multi-stage expert survey (Delphi method), consensus on twelve items for the assessment tool was reached. To validate the assessment tool, a group of volunteer doctors and medical students performed a POCUS examination using simple linear probe and more complex sector probe techniques. The examination was evaluated by two independent assessors using the created assessment tool. Then, four experts evaluated anonymized recordings of the examinations. We tested the reliability and validity, including internal consistency. Results: A total of 70 examinations were included. Of these, 19 examinations were carried out by physicians and 51 by medical students. A high inter-rater reliability (Cohen’s kappa 0.78 (linear weighted; SEM 0.37; *p* < 0.001) and Krippendorff’s alpha 0.895) was shown for the evaluation tool. To improve discriminative power and strengthen reliability, the assessment tool was modified using Cronbach’s alpha. Modification resulted in the removal of three items (patient positioning, ultrasound mode selection, and probe selection) from the tool. The mean values of instrument and expert ratings were now 2.62% apart (46.90% instrument vs. 44.29% expert). Pearson’s correlation coefficient between tool and expert ratings showed moderate to high validity (r = 0.69; *p* < 0.001). Conclusions: The new assessment tool is highly reliable and a valid tool for assessing POCUS skills. It holds strong potential for integration into medical education and training to objectify ultrasound skills. Further studies are required to investigate discriminatory power and transferability to other POCUS algorithms.

## 1. Introduction

Point-of-care ultrasound examination (POCUS) is becoming a standard procedure in emergency, intensive care, and perioperative medicine [1,2]. POCUS has even been incorporated into current clinical guidelines and recommendations [3,4,5]. POCUS algorithms usually address specific organs or problems, like lung evaluation with bedside lung ultrasound (BLUE) [6], cardiac function assessment with focused echocardiography (FATE) [7], or the presence of free fluid in body cavities using focused assessment with ultrasound in trauma (FAST). Longer algorithms like rapid ultrasound for shock and hypotension (RUSH) [8] try to narrow down multiple causes for one symptom. Different POCUS examinations impose varying demands on the technical and intellectual competencies of the examiner. However, it is still not well understood which specific abilities are most crucial for competent POCUS performance [9]. While becoming a proficient FAST examiner likely does not take much more than a brief introduction and about 30 to 100 examinations [10], performing a sufficient cardiac ultrasound using the FATE protocol is considerably more demanding [7].

While certain POCUS algorithms have been standardized, teaching concepts—and especially assessment tools—lack such standardization. For example, Ramgobin et al. point out that a proper curriculum for POCUS in internal medicine still has to be developed, and proper assessment tools might help broaden POCUS education across different specialties [11]. Also, the existing approaches to quantifying performance in POCUS are focusing specifically on FAST and TTE techniques [12,13]. Established frameworks treat technical image acquisition and interpretation as separate skill sets [14].

The objective of this study was to develop and validate an assessment tool that evaluates an ultrasound examiner’s manual and technical skills to acquire interpretable images. Rather than targeting a specific POCUS algorithm, we aimed to cover a broad range of technical competencies across different examination techniques. Image interpretation and clinical decision-making represent distinct competencies that require separate assessment algorithms and were therefore not included in this study.

## 2. Methods

### 2.1. Study Design

**Figure 1:**
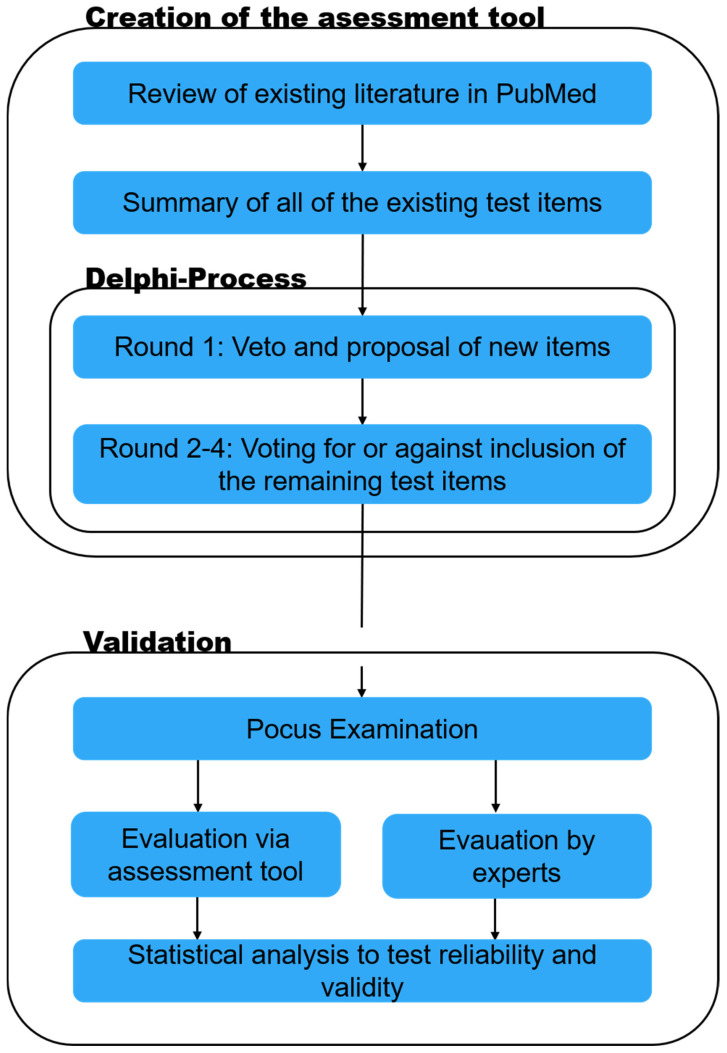
Study design. The study consists of two parts. Part one is the creation of the assessment tool through literature research and the Delphi process, and part two is the application and validation of the developed assessment tool.

For the creation of the assessment tool in this single-center observational study, a literature review was conducted to identify suitable items. These items then underwent a Delphi process for the creation of the assessment tool. Secondly, the tool was used to grade POCUS performers and tested for its reliability and validity (Figure 1).

#### 2.1.1. Delphi Process and Creation of the Assessment Tool

A review of the existing literature was performed exclusively on PubMed by searching for “POCUS [and] assessment tool”; 187 results were identified. The search period covered the past ten years (January 2012–December 2022). Only six studies from 2016 until 2022 addressed assessment of POCUS users and were found suitable [14,15,16,17,18,19]. All of them were published in English.

Out of these six studies, 17 potential items were identified for the new assessment tool (Table 1). An invitation to participate in a four-step Delphi survey was sent to 84 POCUS experts at Saarland University Medical Center. We defined as experts those specialists in anesthesia, intensive care, and emergency medicine who hold certificates from various POCUS courses, e.g., that of the German society for ultrasound in medicine (DEGUM); teach ultrasound to medical students; or serve as instructors for POCUS courses. In the first round, the so-called veto round, the surveyed experts could reject (veto) items they considered unsuitable. They also had the opportunity to suggest additional items. In the following three rounds, the process shifted from vetoing to voting. The surveyed individuals could vote for or against the inclusion of each of the remaining evaluation points. Only experts who had participated in the previous round were invited to subsequent rounds. We conducted the survey online using Microsoft Forms (Microsoft Corp., Redmond, WA, USA). In the first Delphi round, 15 out of 84 invited experts participated. In the second round, all 15 of these experts took part again. In the third round, 10 out of 15 experts participated, and in the fourth round, all 10 of these experts completed the survey. Consensus was defined as at least 80% agreement among participants on each item.

#### 2.1.2. POCUS Examination and Initial Grading

Before the evaluation of the assessment tool, the study was registered in the German Clinical Trials Register (DRKS00033830). After approval by the ethics committee (Ethikkommission der Aerztekammer des Saarlandes, registration 133/23, 13 July 2023 and 13 February 2024) and individual written informed consent, 70 volunteers were included in the study. They were asked to perform a POCUS examination after receiving brief instructions on the ultrasound device and the POCUS algorithm used. We used a custom POCUS algorithm for this study. It involved a bilateral lung ultrasound exam using a linear probe in the intercostal spaces over the upper lobes utilizing B- and M-Mode. This was followed by a short heart ultrasound incorporating parasternal long- and short-axis views, as well as a subcostal view. The combination of more straightforward linear probe techniques and presumably more difficult sector probe approaches seemed to be ideal to comprehensively assess examiner competence. The examiners were allowed to use an instruction sheet that showed the sequence of all ultrasound views required when performing the algorithm (bilateral lung ultrasound views—using a linear probe for B- and M-modes—followed by cardiac views: parasternal long axis, short axis, and subcostal views with a sector probe). Image interpretation skills were not tested. Two observers used the novel twelve-item assessment tool (Table 2) to grade the participants independently. Initially, during POCUS examination, the highest possible score was twelve points, and the lowest possible score was zero points. After data collection, i.e., after POCUS examination, and after reliability testing via Cronbachs’s alpha, the first three items of the assessment tool were removed (Table 2). Thus, nine items remained. The removal did not impair validity.

#### 2.1.3. Examination Setup and Equipment

The 70 examiners performed an ultrasound on the same healthy volunteer who was positioned in the supine position. Ultrasound was performed with Samsung HealthCare HS 50 (Samsung, Suwon, South Korea). There were three ultrasound probes attached: Linear (LA3-14AD), Convex (CA1-7AD), and Phased-Array (PE2-4). To evaluate the handling of the POCUS examiner and the quality of his ultrasound pictures, the whole examination was recorded, and every examiner was filmed by a 4k camera (Panasonic Lumix DMC-G81; Panasonic, Kadoma, Japan) simultaneously. The camera was positioned on a tripod at a height of 160 cm to the left of the healthy volunteer, at the level of the xiphoid, and 20 cm away from the bed. The image was centered on the xiphoid and not magnified. The examiners were allowed to change the position of the ultrasound device and the healthy volunteer on the bed. The filmed videos were edited using Apple’s Final Cut Pro software (Apple, Cupertino, CA, USA) and synchronized side by side. The body and the head of the examiner were blurred. No other adjustments were made to the video material.

#### 2.1.4. Statistical Analysis

Data were collected using Excel 2020 (Microsoft Corp., Redmond, WA, USA). Statistical analysis were performed using SPSS 29.0.1.1 (IBM, Armonk, NY, USA). Data were tested for normal distribution (Shapiro–Wilk) and expressed as means (±SD).

#### 2.1.5. Reliability Testing

To measure the internal consistency of our assessment tool, we calculated Cronbach’s alpha. Cohen’s kappa coefficient was calculated to determine the inter-rater reliability for every item of the assessment tool. For the metrically scaled data of the overall evaluation, Cohen’s kappa was weighted linearly and Krippendorff’s alpha was calculated.

#### 2.1.6. Validation Process

For validation, four experts, who had participated in the first round of the Delphi process, watched videos of the POCUS examinations via Microsoft Stream (Microsoft Corp., Redmond, WA, USA) retrospectively. On the video, it was impossible to identify the examiner due to pseudonymization and a blurred body. The experts were asked to rate the overall performance of the examiner on a scale between 0 (totally insufficient) and 10 (very proficient), based on their subjective impression. They were asked to specifically consider handling of the ultrasound probes, quality of the images generated, and general performance of the examiner. The expert ratings were then correlated with the findings of the observers using the assessment tool. We used Pearson’s correlation coefficient to compare the experts’ evaluation with the results of the assessment tool to determine validity.

## 3. Results

### 3.1. Tool Development and Delphi Process

In the first round, we received 15 responses. Four items were removed from consideration by a majority vote. No new items were proposed by the experts. In the second round of voting, again, 15 of the 84 experts responded. For five items, a consensus was reached. In the third round, the survey was distributed to the 15 participants from the second round. Of these 15 experts, 10 individuals responded and a consensus was reached on four more items. In the fourth round, all 10 experts who had been contacted responded. A consensus was reached on three further items. One remaining item could not reach consensus and was therefore removed. Consequently, the assessment tool consists of a total of twelve items.

### 3.2. Examination Results

Seventy examiners performed ultrasound on the same healthy volunteer. Fifty-one of them were medical students, and nineteen examiners were medical doctors. Results were available for all ultrasound probe positions and from all of the examiners. For the student group, the mean score on the novel assessment tool was 42.4% (±21.6%), with a mean expert performance rating of 39.9% (±14.8%). For the medical doctors, assessment tool mean score was 58.7% (±23.6%), and mean expert rating was 55.9% (±15.0%). We also measured the time taken for the examination as a secondary outcome: it took the students 227 s (±86 s) and the doctors 170 s (±64 s) to perform the exam.

### 3.3. Reliability

Inter-rater reliability between the assessors using the tool was calculated with Cohen’s kappa of 0.778 (SE = 0.37; *p* < 0.001) and Krippendorff’s alpha of 0.895 retrospectively. The assessment tool was further tested for internal consistency. Cronbach’s alpha was calculated for every item of the tool (Table 3). We were able to further increase Cronbach’s alpha by removing the first three items. Those three items showed low separation efficiency (corrected item–scale correlation < 0.3), meaning they were not discriminative enough. We tested if the elimination had any influence on validity and found that the removal had no impact on Pearson’s correlation coefficient and thus no influence on validity.

### 3.4. Validity

**Figure 2:**
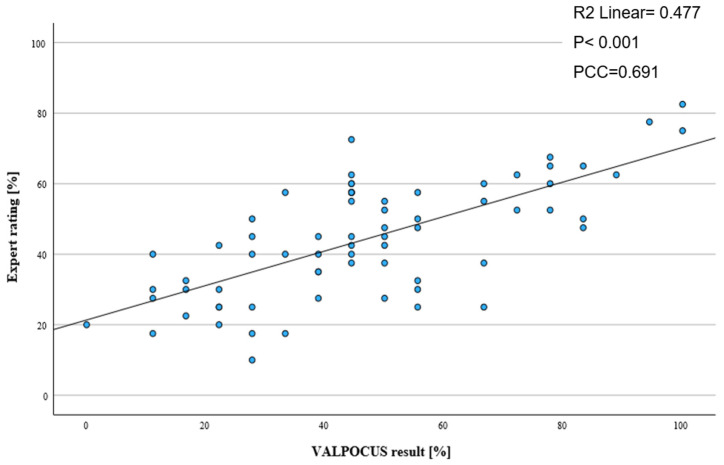
Scatter plot with linear regression of the ratings of the adjusted assessment tool and the expert ratings (PCC: Pearson correlation coefficient).

After adjusting internal consistency of the test, mean ratings of the assessment tool and expert opinion were compared. We were able to show that there was only a minimal difference between overall mean results. For the adjusted assessment tool, we calculated a mean score of 46.9% (±23.18%), and for the expert ratings, the mean score was 44.29% (±16.42%), suggesting reasonable validity. To examine validity, the result of each test performed by an examiner using the tool was correlated with the respective expert rating (Figure 2). Pearson’s correlation coefficient was calculated at 0.691 (R^2^ = 0.477; *p* < 0.001), indicating a moderate correlation between the assessment tool scores and expert ratings.

## 4. Discussion

The development of our 12-item assessment tool, VALPOCUS, was accomplished through a multi-stage Delphi process involving 15 POCUS experts. The tool’s high inter-rater reliability and strong internal consistency, along with the effective separation efficiency of the test items, underscore the success of our experts in selecting appropriate items. Given that VALPOCUS also demonstrated moderate-to-high validity when compared to expert opinion, it appears to be a suitable tool for formally and objectively assessing POCUS skills. In this context, it is worth mentioning that while the R^2^ value of Pearson’s correlation supports the tool’s validity, it is not optimal, indicating moderate-to-strong validity in this context. We attribute this to the subjective nature of the expert evaluations, as they were asked to rate the overall performance without specific guidelines to focus on individual aspects. However, the binary nature of the items do facilitate straightforward adaptation for everyday clinical teaching. For instance, clinicians can use VALPOCUS items to focus on specific abilities that are highly correlated with proficient POCUS performance. On the other hand, the binary format has limitations in capturing different nuances of clinical skills. But since this tool shall provide insights into the manual and technical abilities, the binary format’s advantages, like consistent (simple and objective) grading across different examiners, may justify a lack of depth of assessment for subtle clinical skills.

There is widespread consensus that ultrasound, and especially POCUS, is becoming more and more important in daily medical routine and therefore medical education. Interestingly, to date, only a few high-quality assessment tools in POCUS [13,20] are available. One of these tools is the Ultrasound Competency Assessment Tool (UCAT) [13] by Bell and colleagues. Notably, validation in UCAT was performed by utilizing Messick’s validity framework [21], which correlates UCAT’s test results with surrogates for performer competency. The UCAT results were therefore correlated with the performers’ level of training, number of previously performed scans (which again is a surrogate for ultrasound experience), and their self-rated POCUS confidence. Direct observation was suggested by the authors to further amend validity but was not part of the development process. In our study, the VALPOCUS-graded exams were retrospectively observed and graded by experts involving anonymized video recordings. Therefore, VALPOCUS might be more suitable for objective skill evaluation in standardized POCUS training.

Correlation between these direct observation expert ratings and VALPOCUS results was used to determine validity. Yet, other than the distinction in qualification (“student” and “medical doctor”), which has, according to Bell et al. [14], the weakest correlation to POCUS performance, surrogates were not assessed individually. Eventually, combining the two approaches used in UCAT and VALPOCUS development could possibly provide an even more comprehensive understanding of the validity of individual or combined assessment items. This would be an excellent starting point for future studies.

An individual POCUS algorithm, the combination of lung ultrasound techniques (linear probe) and transthoracic heart ultrasound (sector probe) was chosen for this study. The intention of choosing a non-standardized algorithm over FAST or BLUE was to cover a broader POCUS skill-spectrum and therefore minimize the risk of a potential ceiling effect in the test. This effect occurs when a test fails to differentiate between skill levels because the task does not challenge the participants sufficiently. As a result, the assessment tool may not effectively measure variations in skill, leading to skewed data and reduced validity [22,23]. Even though we are convinced that this measure helped to generate more heterogeneous skill requirements and an improvement in test validity, more studies are needed to determine if its results are transferable to other POCUS algorithms.

VALPOCUS’ reliability and internal consistency were first tested after a pilot phase of 10 exams. As the results of Cronbach’s alpha and Cohen’s kappa coefficient were promising, the study was continued using the 12-item rating tool. However, after all exams were finished and reliability was tested again, we found that the first three items (correct patient position, correct ultrasound mode, and suitable ultrasound probe selected) showed significantly lower item-scale correlations and their removal led to a higher Cronbach’s alpha and therefore better reliability without impairing validity. Therefore, in this study, the first three items were excluded from statistical scoring to improve internal consistency, but they remain part of the conceptual VALPOCUS tool, as they may be of greater importance in other POCUS algorithms. Also, it remains unclear if this effect is caused by true, insufficient discrimination abilities of the first items or if this is related to study design. Particularly because in our algorithm, patient position was meant to be supine and would not change throughout the exam; also, no other ultrasound mode than B- and M-Mode (lung) was required, and probe selection was implicated by sample pictures on the instruction sheet. As the three items were deemed suitable and successfully passed the expert panel during the Delphi process, and because they will very likely be of more importance in algorithms where patient positioning, mode, and probe selection change repeatedly, we decided to keep them in the VALPOCUS algorithm.

### Limitations

The representativeness of our Delphi consensus is limited by the relatively low initial participation rate (15/84 experts) and the single-center design of the study, which may reduce generalizability of the selected items.

We acknowledge that, even though the suggested items were extracted by comprehensive literature research, solely involving experts of our own center in this single-center observational study might have led to a biased selection of the algorithm’s items. In our study, all four validation experts had also participated in the first Delphi round. Although they did not take part in subsequent Delphi rounds, this overlap may have introduced observer expectation bias and potentially influenced the correlation between expert ratings and tool scores. It is worth noting that image interpretation skills and clinical implications of relevant findings are not assessed with VALPOCUS. Thus, it does not specifically address the clinical evaluation of dyspneic patients or the full spectrum of lung and cardiac findings in pathological states. As these aspects are surely relevant in everyday practice, VALPOCUS-based examination should ideally be combined with clinical expertise. Thus, we see the need for future iterations of the tool to incorporate graded scales for more complex skill assessments.

Finally, the unequal distribution between medical students and physicians in our study did not directly affect the validity analysis, which was based on correlations across all participants. Nevertheless, the group imbalance may have influenced the overall performance spectrum and should be considered a limitation when interpreting the results.

## 5. Conclusions

VALPOCUS, a novel assessment tool to evaluate POCUS performers, was developed. In our pilot test, VALPOCUS demonstrated good reliability. Moreover, the tool showed evidence of validity when compared to expert opinion, effectively evaluating performers of a POCUS algorithm that incorporated tasks of varying difficulty in healthy volunteers. Future work should focus on multi-center validation, application in pathological cases, and the integration of higher-level interpretive and decision-making skills.

## Figures and Tables

**Table 1:** Test items after literature research and following the Delphi process.

Responses	Round 1 15/84	Round 2 15/84	Round 3 10/15	Round 4 10/10
Best position of the ultrasound device	**V**			
2. Correct patient position		14/15	**10/10**	
3. Correct ultrasound mode		14/15	**10/10**	
4. Suitable ultrasound probe		14/15	**10/10**	
5. Correct position of the ultrasound probe indicator		10/15	4/10	**No:6/10**
6. Suitable positioning of the ultrasound probe		**15/15**		
7. Correct pressure on the probe	**V**			
8. Correct probe handling	**V**			
9. Correct adjustments for different probes	**V**			
10. Identification of organs and tissue		**15/15**		
11. Identification of artefacts		**15/15**		
12. Identification of wrong images		**15/15**		
13. Centering of the target organ/tissue		12/15	8/10	**10/10**
14. Assessor finds the ultrasound images adequate to evaluate heart and lung		10/15	9/10	**10/10**
15. Using options to optimize the ultrasound angle/gate		13/15	**10/10**	
16. Depth is adjusted		13/15	9/10	**10/10**
17. Gain is adjusted		**15/15**		

In the first round, there was a veto against four items. All remaining items passed the second and third rounds. In the fourth round, Item 5 received less than 80% approval rate and was removed. V: veto; red: item removed; green: consensus reached; numbers express agreement/total answers. In the first round, there was a veto against four items. All remaining items passed the second and third rounds.

**Table 2:** Assessment tool after Delphi process. Internal consistency increased when items 1–3 were removed.

	Assessment Tool	
1	Correct patient position	
2	Correct ultrasound mode	
3	Suitable ultrasound probe	
4	Suitable positioning of the ultrasound probe	
5	Identification of organs and tissue	
6	Identification of artefacts	
7	Identification of wrong images	
8	Centering of the target organ/tissue	
9	Observer/Assessor deems the ultrasound images adequate for evaluation	
10	Performer optimizes the ultrasound image	
11	Depth is adjusted	
12	Gain is adjusted	
	(1 point per item) points:	____/12

**Table 3:** Internal consistency testing using Cronbach’s alpha. For items 1–3, the corrected item scale correlation was < 0.3. Cronbach’s alpha increased if removed. Thus, the internal consistency was increased after their removal. Removal had no impact on Person’s correlation coefficient.

	Cronbach’s Alpha	Corrected Item-Scale-Correlation
Total	0.838	
Item		
1	0.840	0.0
2	0.841	0.021
3	0.840	0.0
4	0.838	0.318
5	0.814	0.534
6	0.820	0.481
7	0.813	0.544
8	0.805	0.603
9	0.815	0.538
10	0.839	0.320
11	0.826	0.428
12	0.823	0.402

## Data Availability

The original contributions presented in this study are included in the article. Further inquiries can be directed to the corresponding author.
